# Time to diagnosis and mortality in colorectal cancer: a cohort study in primary care

**DOI:** 10.1038/bjc.2011.209

**Published:** 2011-06-07

**Authors:** M L Tørring, M Frydenberg, R P Hansen, F Olesen, W Hamilton, P Vedsted

**Correction to**: *British Journal of Cancer* (2011) **104**, 934–940; doi:10.1038/bjc.2011.60

In the original, published Figure 2, patients were grouped incorrectly: (**A**) showed patients with alarm symptoms of cancer, and (**B**) showed patients with symptoms related to any serious illness together with patients with vague symptoms not directly related to cancer or any other serious illness. The reported numbers of subjects and deaths were not consistent with the incorrect grouping. The mistake was graphical and does not in any way alter the conclusions of the paper. The corrected Figure 2 is shown below.

## Figures and Tables

**Figure 2:**
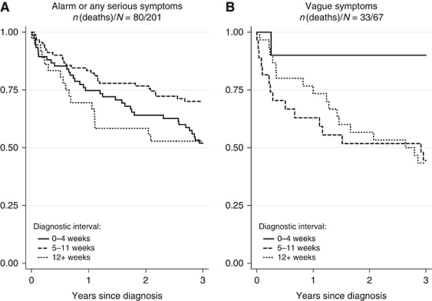
Estimated survival according to the length of diagnostic interval analysed for colorectal cancer patients presenting with (**A**) alarm symptoms of cancer or symptoms related to any serious illness and (**B**) vague or ill-defined symptoms not directly related to cancer or any other serious illness. The solid curves indicate 0–4 weeks; dashed curves indicate 5–11 weeks; and dotted curves indicate ⩾12 weeks from first presentation of symptoms in primary care to diagnosis (the diagnostic interval).

